# Cytotoxicity of Polyaniline Nanomaterial on Rat Celiac Macrophages *In Vitro*

**DOI:** 10.1371/journal.pone.0107361

**Published:** 2014-09-24

**Authors:** Yu-Sang Li, Bei-Fan Chen, Xiao-Jun Li, Wei Kevin Zhang, He-Bin Tang

**Affiliations:** Department of Pharmacology, College of Pharmacy, South-Central University for Nationalities, Wuhan, PR China; East Carolina University, United States of America

## Abstract

Polyaniline nanomaterial (nPANI) is getting popular in many industrial fields due to its conductivity and stability. The fate and effect of nPANI in the environment is of paramount importance towards its technological applications. In this work, the cytotoxicity of nPANI, which was prepared by rapid surface polymerization, was studied on rat celiac macrophages. Cell viability of macrophages treated with various concentrations of nPANI and different periods ranging from 24 to 72 hours was tested by a MTT assay. Damages of nPANI to structures of macrophages were evaluated according to the exposure level of cellular reactive oxygen species (ROS) and change of mitochondrial membrane potential (MMP). We observed no significant effects of nPANI on the survival, ROS level and MMP loss of macrophages at concentrations up to 1 µg/ml. However, higher dose of nPANI (10 µg/ml or above) induced cell death, changes of ROS level and MMP. In addition, an increase in the expression level of caspase-3 protein and its activated form was detected in a Western blot assay under the high dose exposure of nPANI. All together, our experimental results suggest that the hazardous potential of nPANI on macrophages is time- and dose-dependent and high dose of nPANI can induce cell apoptosis through caspase-3 mediated pathway.

## Introduction

Nano-conductive-polymer materials develop rapidly in recent years. They have been widely applied in chemical, electronic, aerospace and medical field thanks to the combined excellent characteristics of both nanomaterial and conductive polymer. Along with the increasing production and use of nanomaterials, there has been a growing concern about the hazardous of inevitable unintended human exposure [1]–[3]. These nanometer-sized materials can access, mostly through the lungs, gastrointestinal tract, skin, injection and implantation, into human body posing significantly serious health threats [4]–[6].

As one of the most famous conductive polymer, nano-structured PANI (nPANI) was rapidly developed in industries and clinical fields with a lot of advantages, including availability of its raw materials, easy preparation, maneuverability of particle size and morphology, good electrical conductivity, environmental stability, biocompatibility and the reversible doping-dedoping process [7]–[10]. Recently, limited researches [11]–[14] had been carried out on the biocompatibility and cytotoxicity of PANI and its composites, but there was no definite answer whether the use of nPANI is safe or not. Therefore, the uncertainty of its toxicity is still a big problem for wide range of bioapplications [15].

Macrophages are important cells of the immune system that are responsive to cell debris and pathogens. Its applicability in immuno-toxicology has been established for cytotoxicity of exogenous chemicals [16]. In the present study, nPANI was prepared in the form of nano fibers by the rapid surface polymerization process [17]–[19]. Its cytotoxicity and toxic mechanism on rat celiac macrophages were examined by cell death, cellular reactive oxygen species (ROS) level, loss of mitochondrial membrane potential (MMP) and apoptosis-associated caspase activation.

## Materials and Methods

### Preparation of nPANI

Typically, nPANI was prepared with a surface polymerization method, by using ammonium persulfate (APS; Sinopharm, China) as the oxidant and hydrochloric acid as the doping agent. The procedures were described in details in our previous report [20]. After polymerization, the prepared neutral nPANI solution was then further dialyzed by saline to remove hypertoxic oligomer. The well cleaned nPANI was stocked in the form of neutral aqueous suspension (15 mg/ml) prior to use.

### Characterization of nPANI

The morphology of nPANI was observed with a scanning electron microscope (SEM, Hitachi S-4800, Japan). The UV-Vis absorption spectra of nPANI suspensions were measured on a Varian Cary 50 UV-Vis spectrophotometer (Varian Cary 50, Agilent). The Fourier infrared spectra of nPANI were recorded on a Bruker VERTEX 70 infrared spectrometer in a transmission mode.

### Animal care

The care and use of animals for this study were performed according to the Guide for Animal Experimentation, South-Central University for Nationalities and the Committee of Research Facilities for Laboratory Animal Sciences, South-Central University for Nationalities, China. The protocols were approved by the Committee on the Ethics of Animal Experiments of the South-Central University for Nationalities, China (Permit Number: 2011-SCUEC-AEC-002). All efforts were made to minimize suffering.

### Cell culture

The isolated primary macrophages from adult Wistar rat abdominal cavity (6–9 weeks of age) were maintained in Dulbecco’s-modified eagle’s medium (DMEM, Gibco, USA) containing 10% (v/v) fetal bovine serum (Gibco, USA), 1% penicillin-streptomycin solution, and suspended to a density of 6.0×10^5^ cells/ml. Cells were seeded in a 96-well plates with 200 µl per well. Eight wells were supplied for each exposing nPANI concentration, three of them were used as background correction, and the rest were experimental group. Cells were allowed to attach for 12 hours onto the 96-well plate under 5% CO_2_ at 37°C. Then, they were exposed with regular growth medium containing nPANI at different concentrations ranging from 0.1 to 100 µg/ml or pure water as a negative control unless otherwise instructed.

### Cell morphology and viability assays

Cell viability was measured by a MTT assay. After being exposed to different concentrations of nPANI for 24 to 72 hours, cells were imaged by an inverted phase contrast microscope (Nikon Eclipse Ti-S, Japan). Then cells were rinsed with PBS and recovered by incubating with fresh culture medium without serum for an hour. After that, 5 mg/ml MTT (3-(4,5-dimethylthiazol-2-yl)-2,5-diphenyltetrazolium bromide, Sigma, USA) was added directly to the medium. After adding MTT, all samples were incubated for 4 hours at 37°C. Medium was then removed from cells, and DMSO was added to the wells to dissolve the formazan crystals. The absorbance was measured at 490 nm after oscillation of 15 minutes. All of the experiments were performed in triplicates. The results were expressed as percentages of the control (without nPANI).

### Reactive oxygen species (ROS) assay

The generation of superoxide radical and hydrogen peroxide were detected by 2,7-dichlorodihydrofluorescein diacetate (DCF-DA) staining. For ROS assay, 6.0×10^5^ cells per well were cultured in 35 mm dishes, and incubated with nPANI at different concentrations for 6 hours. Then ROS detection kit (Beyotime, China) was used for the assay. ROSup (50 µg/ml) was used as the positive control. Fluorescent intensity was detected by Enzyme-labeled meter (TECAN infinite M200, Switzerland) at an excitation wavelength of 488 nm and an emission wavelength of 525 nm.

### Mitochondrial membrane potential assay

JC-1 fluorescent probe was used to determine the mitochondrial membrane potential change. Cells in this assay were cultured as the former. Cells treated with carbonyl cyanide 3-chlorophenylhydrazone (CCCP; 0.01 mM) were considered as the positive control. All the processes were conducted following the test kit (Beyotime, China). Fluorescent intensity of JC-1 monomer was detected by Enzyme-labeled meter (TECAN infinite M200, Switzerland) at an excitation wavelength of 490 nm and an emission wavelength of 530 nm, while that of JC-1 polymer was detected at an excitation wavelength of 525 nm and an emission wavelength of 590 nm. In this study, the ratio of JC-1 monomers and polymers was used as a representation of MMP (mitochondrial membrane potential).

### Western blot assay

After incubating with control, 10 and 100 µg/ml nPANI for 24 hours, cells were lysed with protein lysis buffer (Beyotime, China). The concentration of total protein was detected by Lowry method. Equal amounts of protein were fractionated by 10% SDS gels and transferred to polyvinylidene difluoride membranes (Millipore Corporation, USA). After blocked with 5% nonfat dry milk, the membranes were incubated with an anti-caspase3 antibody (1∶1000 dilution, Boster, China) or anti-actin antibody (1∶1000 dilution, Boster, China) at 4°C overnight. After removing primary antibodies, the membranes were washed 3 times for 5 minutes by TBST (Tris-Buffered Saline, 0.1% Tween-20) solution and followed by exposure of secondary antibodies (1∶5000 dilution, goat anti-rabbit or goat anti-mouse, Boster, China) for 2 hours at 4°C. Finally, after wash, bands on the membranes were visualized by developer and fixing solution [21]. The protein bands were quantified using the ImageJ software (NIH).

### Statistical analysis

One-way analysis of variance (ANOVA) was used as the statistical test. Results shown in the figures are expressed as means ± standard error of mean (SEM). A value of *P*<0.05 was considered statistically significant.

## Results

### Characterization of nPANI

The nPANI was prepared by oxidative polymerization of aniline, resulting in the production of a mixture containing polymer, aniline oligomer, diphenylamine and benzidine. The polymerization was initiated by adding APS into aniline solution. Once started, the solution turned from initial colorless to weak green, and finally to dark blue. After the polymerization, the nPANI product was cleaned by dialysis, and the suspension was neutralized with diluted ammonia water, resulted in a pure neutral water solution.

**Figure 1:**
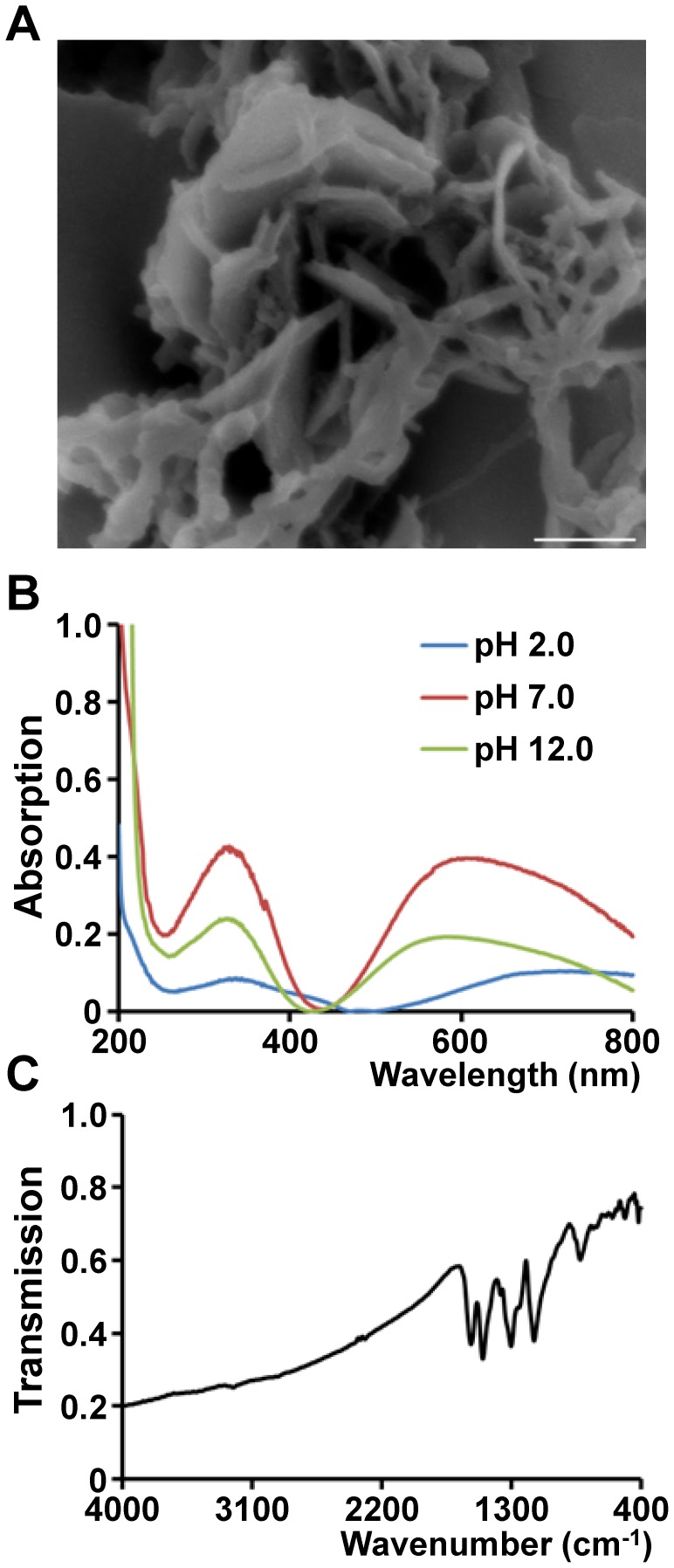
Characterization of nPANI. (A) The scanning electron microscope image of nPANI. Scale bar is 500 nm. (B) UV-vis absorption spectra of nPANI aqueous solutions at pH 2.0, pH 7.0, and pH 12.0. Ultra DI-water was used as a background solution. (C) FTIR spectrum of nPANI. KBr was used as a background.

Scanning electron microscopy revealed the morphology of polymerized nPANI, as shown in Fig. 1A. Polymers presented a typical nanofiber structure, being in consistent with the previous report [20]. Fig. 1B showed the UV-vis absorption spectra of nPANI aqueous solutions at different pHs. At acidic pH, nPANI is in its acid doped state. The absorptions of acid doped PANI at pH 2 were located at 350 and 750 nm, which are attributed to π-π* and π-polaron transitions, respectively. This suggested that the nPANI material at pH 2 had a higher level of proton doping [22]. When the suspension was adjusted to neutral or alkaline, the aforementioned bands were blue shifted to 330 and 580 nm at pH 7, and 330 and 550 nm at pH 12, respectively, representing the dedoping of nPANI. The state of nPANI changed from emeraldine salt to emeraldine base during such a dedoping process, which is consistent with the observation for conventional PANI reported by Wan and Yang [23].

Fig. 1C showed the FTIR spectrum of nPANI, where the characteristic bands of PANI were observed [24], [25]. For example, the C = C stretching vibration of quinoid, C = C stretching vibration of benzene rings, C-N stretching of secondary aromatic ring, and out-of-plane bending vibrations of C-H occurred at 1580, 1498, 1303 and 828 cm^−1^, respectively.

### Cell damage and proliferation

To investigate the cytotoxicity of nPANI, rat celiac macrophages were selected. Cell death, loss of mitochondrial membrane potential (MMP), cellular reactive oxygen species (ROS) level and apoptosis-associated caspase activation were quantified in the present study.

**Figure 2:**
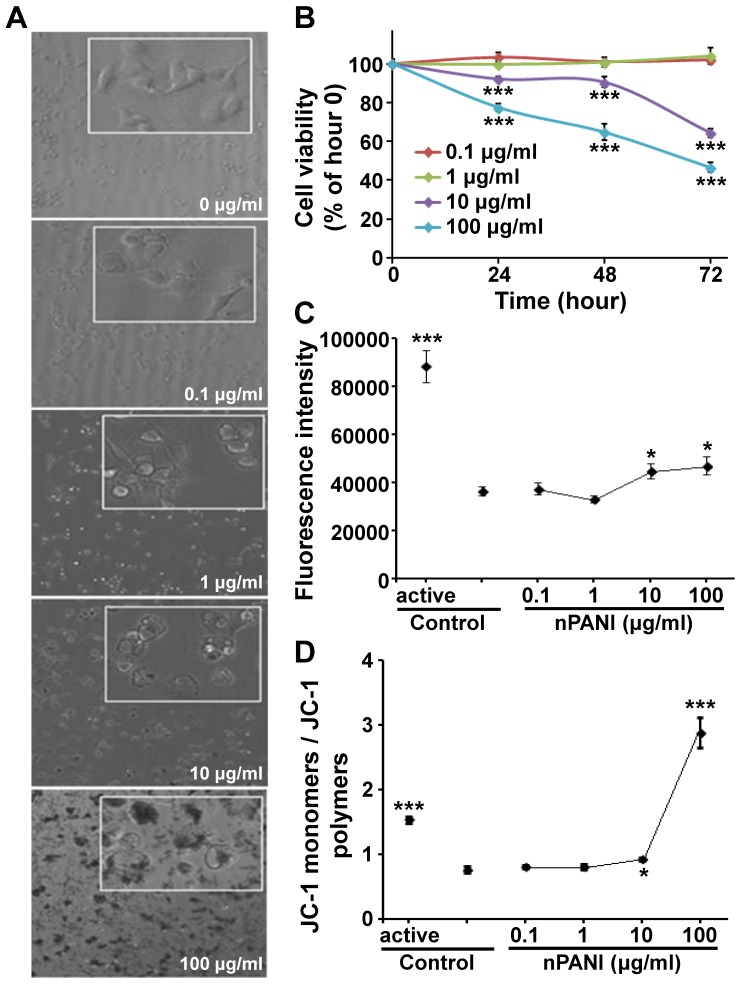
Cytotoxicity of nPANI. (A) Morphology of macrophages under phase-contrast microscope (20× objective, inset: 40×) treated with nPANI at different concentrations. (B) Cell viability of macrophages treated with nPANI at different concentrations for different time periods. (C) ROS production by macrophages after being incubated with nPANI at different concentrations for 6 hours. (D) Effect of 6 hours incubation of nPANI on the mitochondrial membrane potential of macrophages. All data were expressed by mean ± S.E.M. of six measurements and each experiment was performed in triplicate. * and *** denote *p*<0.05 and 0.001 versus the corresponding control.

As we know, morphological features played a leading role in the description of cell death. In Fig. 2A, the control macrophages adherent to the culture dish firmly with clear and smooth contour. The density and morphological features of cells exposed in 0.1 and 1 µg/ml nPANI for 24 hours showed no significant difference from the control group. However, in groups treated with 10 and 100 µg/ml nPANI, the density of cells was less and the cells were more transparent with vaguer rims compared with the control group. Moreover, vacuoles of different sizes could be visualized in these cells, and we also noticed tiny blue particles in the vacuoles.

Next, we quantified the cytotoxicity of nPANI on the macrophages, using concentrations of 0.1–100 µg/ml and treatment time of 24–72 hours. As shown in Fig. 2B, the cytotoxicity of nPANI on macrophages was dose- and time-dependent. When the concentration of nPANI was less than 1 µg/ml, cell viability remained to be 100% (*P*>0.05) in 72 hours. However, when the concentration went beyond 10 µg/ml, cell apoptosis existed in 24 hours after the incubation of nPANI (*P*<0.05, *P*<0.001, respectively). With the exposure time extended, more cells were dying in these two groups. In one word, the cytotoxicity of nPANI is dose- and time-dependent.

### ROS generation

Environmental stress, such as UV exposure or heat, can increase ROS levels dramatically due to significant damage to cell structures. Therefore, to characterize the cytotoxicity of nPANI, ROS production of cells was also monitored. In Fig. 2C, hardly any change of ROS production was detected in low concentration (less than 1 µg/ml) treated group, while in the ROSup (positive control) treated group a huge increase was seen (*p*<0.001). Consistent with the cell viability assay, nPANI with concentration more than 10 µg/ml could promote intracellular active oxygen level and hence induce oxidative stress reaction and cell death.

### Mitochondrial depolarization

Mitochondrial depolarization impairs the efficacy of the electron transport chain leading to massive ROS production. In order to further characterize the cytotoxicity of nPANI, we measured mitochondrial membrane potential (MMP) of cells. As shown in Fig. 2D, After 6-hour incubation, the ratio of JC-1 monomers to polymers in macrophages exposed in 0.1 and 1 µg/ml nPANI was not significant compare with the control group, suggesting that low concentration of nPANI could not alter MMP level of macrophages. However, 10 and 100 µg/ml nPANI could significantly change the mitochondrial membrane potential of macrophages.

### Increased caspase-3 expression

**Figure 3:**
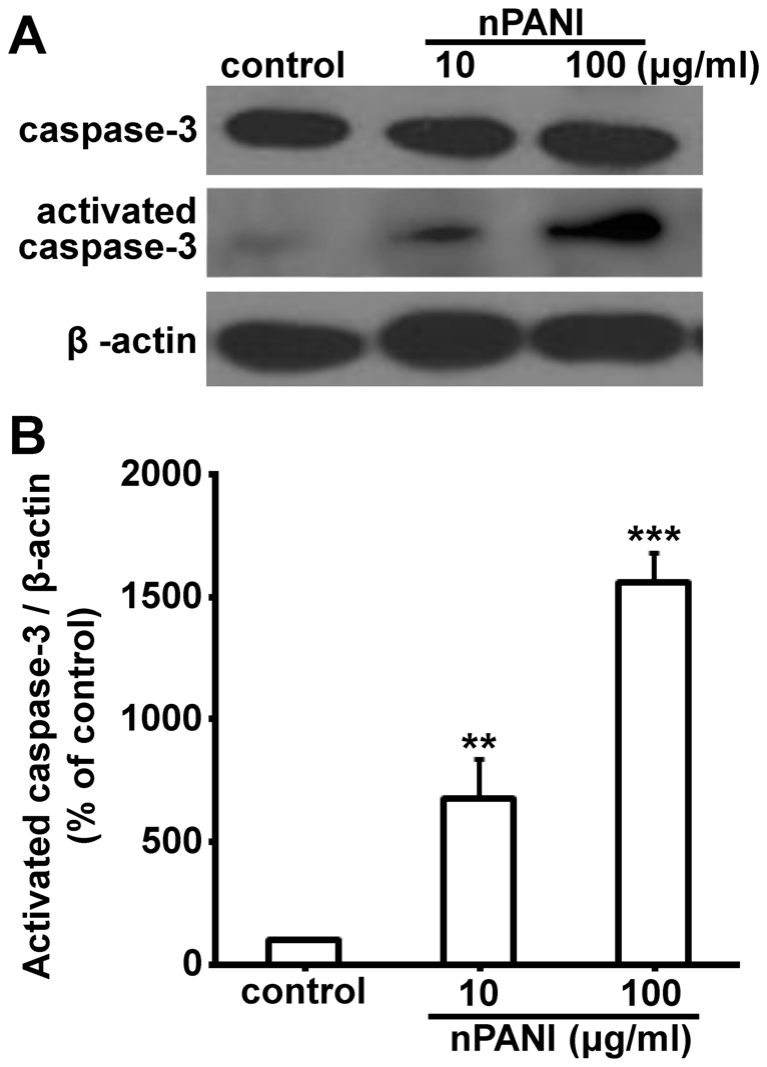
The effects of nPANI on the expressions of caspase-3 and activated caspase-3 expressions in macrophages. (A) Representative Western blots of the caspase-3 and β-actin expressions. (B) The relative levels were analyzed by determining the ratio of the activated caspase-3/β-actin. The values are the means ±S.E.M. of three replicates. ** and *** denote *p*<0.01 and 0.001 versus the control, respectively.

Since high dose nPANI could induce cell apoptosis, we examined the expression and activation of caspase-3, an apoptosis related protein, under such condition. In Fig. 3, high dose nPANI (10, 100 µg/ml) induced more expression of caspase-3 protein in macrophages 24 hours. In addition, activated caspase-3 could be observed, suggesting that caspase-3 was activated to different degrees in both treated groups. Therefore we speculated that caspase-3 might play a regulatory role in nPANI induced apoptosis of macrophages.

## Discussion

In the present study, we have explored the toxicity of nPANI at different concentrations (0.1∼100 µg/ml) on macrophages. The results showed that when concentration of nPANI was higher than 10 µg/ml, macrophage’s survival was significantly threatened, and the harm was dose- and time-dependent. In general, expose of low concentration of nPANI did not show serious toxicity on macrophage in our research, which is consistent with the results of Yslas et al [13]. However, the upper limit of safety threshold of nPANI towards macrophages (less than 10 µg/ml) is lower than that towards lung fibroblast cells (25 µg/ml) [14], suggesting a necessity of studying cytotoxicity of nPANI towards different cell types.

It has been demonstrated that biological effects (such as eukaryotic cell toxicity, anti bacteria effect, and light toxicity of Lolium perenne) of nanomaterials (all kinds of nanometer metal oxide and carbon nanomaterials) are mediated by ROS [26]. Since nanomaterials are usually conductors or semi-conductors, their electric charge would interfere with electronic transduction of cells and thus increase the intracellular level of ROS and affect the permeability of cell membrane. As a result, these nanomaterials would enter cells, accumulate to form a vicious spiral, and destroy cell’s osmotic balance [11]. Finally, the normal function of cells is absolutely restricted, resulting in cell lysis or apoptosis. As shown in Fig. 2C, we have shown that oxidative stress responses in macrophages were elevated when the nPANI concentration go beyond 10 µg/ml.

In order to maintain a certain level of membrane potential, the permeability of mitochondrial membrane to outer electrolyte and foreign material is usually limited. From the early observations, we could clearly see that the macrophages swallow extracellular polyaniline nanoparticles. Microscopic examination also showed that when the nPANI concentration was more than 10 µg/ml, granular nPANI was visible in cells. This led to a speculation that nPANI could contact mitochondria directly after entering into cytoplasm and made the mitochondrial membrane porous, resulting in an increased permeability and unbalanced electrolytes inside the mitochondria. Therefore, the mitochondrial membrane potential would be lost and hence destroy the normal mitochondrial function, and cut off the respiratory chain. As a result, there would be peroxide accumulation in mitochondria and cytosol and cause oxidative stress reaction [27], [28]. In addition, too-low mitochondrial membrane potential would bring about DNA fragmentation, eventually leading to cell death [29]. In accordance with this notion, the mitochondrial membrane potential showed decrease when the concentration of nPANI exceeded 10 µg/ml.

Caspase-3 is an important member of the caspase family. It is expressed widely in different tissue and cell types, and plays a crucial role in the final steps of cell apoptosis [30]. Here we have shown that the expression of active form of Caspase-3 was increased under the treatment of 10 and 100 µg/ml nPANI in a dose-dependent manner, further confirming the involvement of cell apoptosis process in nPANI induced cytotoxicity.

## Conclusion

In this report, we have prepared nanometer-sized organic polyaniline, and this nPANI appeared to cause cytotoxicity on rat celiac macrophages in a time- and dose-dependent manner. We also demonstrated that the cytotoxicity of nPANI was triggered by generation of oxidative stress and change of intracellular mitochondrial membrane potential. In addition, we have shown that the pro-apoptotic protein caspase-3 took part in the nPANI-induced cell apoptosis. All together, we conclude that the safety upper limit for nPANI is less than 10 µg/ml.
